# Bacterial and Protistan Community Variation across the Changjiang Estuary to the Ocean with Multiple Environmental Gradients

**DOI:** 10.3390/microorganisms10050991

**Published:** 2022-05-09

**Authors:** Xinjun Jiang, Zhu Zhu, Jinnan Wu, Ergang Lian, Dongyan Liu, Shouye Yang, Ruifeng Zhang

**Affiliations:** 1 School of Oceanography, Shanghai Jiao Tong University, Shanghai 200030, China; wilming@sjtu.edu.cn (X.J.); wujinnan@sjtu.edu.cn (J.W.); ruifengzhang@sjtu.edu.cn (R.Z.); 2 State Key Laboratory of Marine Geology, Tongji University, Shanghai 200092, China; eglian@tongji.edu.cn (E.L.); syyang@tongji.edu.cn (S.Y.); 3 State Key Laboratory of Estuarine and Coastal Research, East China Normal University, Shanghai 200241, China; dyliu@sklec.ecnu.edu.cn; 4 MNR Key Laboratory for Polar Science, Polar Research Institute of China, Shanghai 200136, China

**Keywords:** bacterial and protistan community, Changjiang Estuary, salinity, alpha diversity

## Abstract

Plankton microorganisms play central roles in the marine food web and global biogeochemical cycles, while their distribution and abundance are affected by environmental variables. The determinants of microbial community composition and diversity in estuaries and surrounding waters with multiple environmental gradients at a fine scale remain largely unclear. Here, we investigated bacterial and protistan community assembly in surface waters from 27 stations across the Changjiang Estuary to the ocean, with salinity ranging from 0 to 32.1, using 16S rRNA and 18S rRNA gene amplicon sequencing. Statistical analyses revealed that salinity is the major factor structuring both bacterial and protistan communities. Salinity also acted as a significant environmental determinant influencing alpha-diversity patterns. Alpha diversity indices for bacterial and protistan communities revealed a species minimum in higher-salinity waters (22.1–32.1). Contrary to the protistan community, the highest bacterial diversity was identified in medium-salinity waters (2.8–18.8), contrasting Remane’s Artenminimum concept. The distribution of major planktonic taxa followed the expected pattern, and the salinity boundary for Syndiniales was specifically identified. These findings revealed the significant effects of salinity on the microbial community across an estuary to ocean transect and the distinct response to salinity between bacterial and protistan communities.

## 1. Introduction

Unicellular plankton, bacteria and protists have central roles in marine food webs and biogeochemical processes, while the distribution, community composition and diversity of microbial communities are determined by both environmental variables and dispersal processes [1,2,3]. Global surveys of bacterial diversity have demonstrated that salinity is the major environmental determinant of bacterial community composition [4]. Besides nutrients, other environmental factors such as temperature, dissolved oxygen and pH are known to determine microbial community structure in estuarine and marine environments [5,6,7,8,9].

A large amount of freshwater and nutrients is delivered into the ocean with river discharge, creating a large environmental gradient from the river to open ocean, which has great impacts on the microbial community composition [10,11,12], gene expression and metabolic profiles [13]. Salinity, nutrients and other environmental variables may affect the community structure of microorganisms [7,14,15]. However, salinity increases along a river-to-ocean gradient coupled with a decrease in nutrients, resulting in the collinearity relationship between salinity and nutrients [16,17]. Therefore, it is necessary to distinguish whether salinity or other factors are the primary factor to determine the microbial community structure.

Studies on community alpha diversity along the salinity gradient across the river-to-sea continuum have been conducted for a long time. Remane’s ‘Artenminimum’ concept reported a minimum of benthic macrofaunal species richness at intermediate salinity levels [18]. Herlemann et al. (2011) first challenged Remane’s ‘Artenminimum’ concept and reported that the Shannon diversity index of bacterial community did not change markedly along the salinity gradient (0–30) in the Baltic Sea [19]. However, conflicting results have been observed in the Baltic Sea: protistan plankton has a species maximum in the freshwater–marine transition zone [20], while no clear trends in alpha diversity for bacterial or eukaryotic communities could be detected along the salinity transect [21]. Moreover, contrary observations were reported from a distinct river–ocean continuum [10,11]. Although previous studies revealed that alpha diversity of the microbial community varied along the river-to-ocean transect, the conclusions were inconsistent between bacteria and protists, and in different river-to-sea continuums.

Bacteria and protists play a significant role in marine food webs and global biogeochemical cycles through different trophic modes. Both unicellular bacteria and protists include some photoautotrophs from river-to-ocean continuums; Bacillariophyta, Cryptophyta and Chlorophyta are dominant primary producers in the river plume [22,23,24]; while Cyanobacteria dominate primary production in the ocean [25]. Energy and nutrients are propagated to heterotrophic protists through grazing and parasitism, while heterotrophic bacteria take up particulate organic matter and decompose particle organic matters. Whether different trophic modes between bacteria and protists lead to their divergent response of community structure to environmental differences in the same river-to-sea gradients remains unclear.

The Changjiang (Yangtze) River is one of the largest rivers in the world, delivering a substantial amount of fresh water into the East China Sea (ECS) and the Yellow Sea every year, with an annual mean runoff of 9.0 × 10^11^ m^3^ [26,27]. With growing populations and rapid industrialization in the Changjiang River Basin, the concentrations of nitrate and phosphate in the Changjiang River have increased significantly over the past 50 years, due to increased industrial wastewater and excessive use of fertilizers in agriculture [27], and approximately 1.43 × 10^6^ t of DIN and 2.35 × 10^5^ t of TP are annually transported to the ocean [28,29,30]. Moreover, about 3.17 × 10^6^ t of DSi is transported into the Changjiang Estuary and adjacent waters every year [31]. These essential nutrients carried by the Changjiang River provide an important source for primary productivity in the marine ecosystem of the adjacent sea [32]. The high flux of organic matter from the Changjiang River affects the biogeochemical cycles in the Changjiang Estuary regions [33]. Moreover, the Changjiang Estuary and its adjacent areas are influenced by multiple water masses from different directions [34]. Previous studies revealed that salinity, temperature, nutrients, dissolved oxygen and other environmental factors regulate the planktonic microbial community composition in the Changjiang Estuary and its adjacent waters [5,35]. However, the primary determinant of microbial community composition in this complex environment has not been clarified.

In this study, the bacterial and protistan communities were investigated using 16S rRNA and 18S rRNA gene-based high-throughput sequencing technology, and the connections between the organisms and the environment were explored. The study aims to address the following questions: (i) Considering a variety of environmental factors, is salinity the most important controlling factor for the microbial community structure in the surface water across the Changjiang river-to-ocean gradient? (ii) How does alpha diversity and community composition of the microbial community change across the Changjiang river-to-ocean gradient? (iii) Are there any differences in the influence of salinity and other environmental factors on the community structure of bacteria and protists?

## 2. Materials and Methods

### 2.1. Sampling

**Figure 1:**
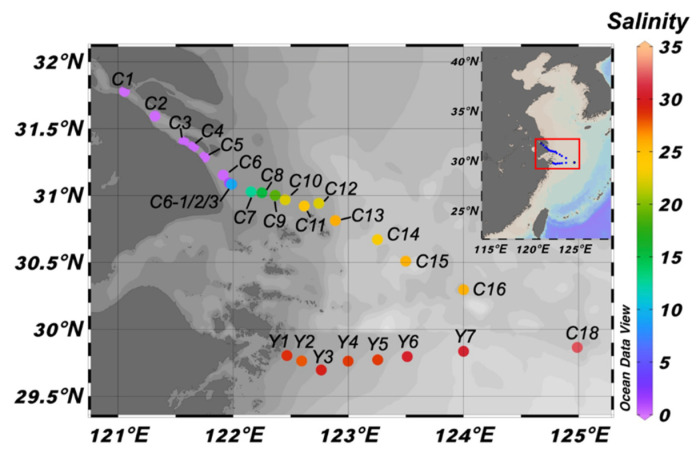
Map of the sampling stations in the Changjiang Estuary and its adjacent waters color-coded according to surface water salinity.

Samples were collected from Changjiang Estuary during the cruise of KECES-2019 (from 28 August to 4 September 2019) onboard the R/V Zheyuke II at 27 stations, including transect C (20 samples) and transect Y (7 samples) (Figure 1). Surface (0.5–2 m) water was collected by a peristaltic pump. Water samples for nutrient determination were directly stored in bottles and placed in a freezer for later analysis. At each station, 0.3 to 4 L of water were immediately filtered onto a 0.2 μm pore-sized membrane filter (Pall Life Sciences, Port Washington, NY, USA) for later biological community analysis. Individual filters were placed in centrifuge tubes and immediately stored at −20 °C. Filters were then transferred at −80 °C to the laboratory and stored at −80 °C until DNA extraction.

### 2.2. Environmental Parameters Measurements

For each sample, temperature and salinity were recorded in situ with the conductivity–temperature–depth (CTD) sensors (SBE-911 Plus, Seabird). Dissolved inorganic nitrogen (the sum of NO_3_-N, NO_2_-N and NH_4_-N), dissolved inorganic phosphorus (DIP), dissolved silicate (DSi), suspended particulate matter (SPM) and Chlorophyll *a* (Chl *a*) were measured by Xu et al. [36]. Surface water pH values were measured on board with a PHS-25 pH meter (±0.01; Shanghai INESA Scientific Instrument Co., Ltd., Shanghai, China). The detailed information of each station is reported in Table S1.

### 2.3. DNA Extraction, PCR Amplification, and High-Throughput Sequencing

OMEGA Soil DNA Kit (D5625-01) (Omega Bio-Tek, Norcross, GA, USA) was used to extract the total genomic DNA according to the manufacturer’s instructions. The quantity and quality of extracted DNA was measured using a NanoDrop ND-1000 spectrophotometer (Thermo Fisher Scientific, Waltham, MA, USA) and agarose gel electrophoresis, respectively.

The V3-V4 regions of the bacterial 16S rRNA genes were amplified using the forward primer 338F (5′-ACTCCTACGGGAGGCAGCA-3′) and the reverse primer 806R (5′-GGACTACHVGGGTWTCTAAT-3′). The V4 region of the protistan 18S rRNA genes were amplified using the forward primer 547F (5′-CCAGCASCYGCGGTAATTCC-3′) and the reverse primer V4R (5′ACTTTCGTTCTTGATYRA-3′) [37]. Sample-specific 7 bp barcodes were incorporated into the primers for multiplex sequencing. PCR amplification was carried out in triplicate. Each 25 μL PCR reaction contained 10 ng of template DNA, 1.25 U of FastPfu DNA Polymerase and a final concentration of 1 × FastPfu buffer, 5 nmol of dNTPs, and 10 nmol of each forward and reverse primer. PCR amplification consisted of an initial denaturation at 98 °C for 5 min, followed by 25 cycles consisting of denaturation at 98 °C for 30 s, annealing at 53 °C for 30 s, and extension at 72 °C for 45 s, with a final extension at 72 °C for 5 min. PCR amplicons were purified with Vazyme VAHTSTM DNA Clean Beads (Vazyme, Nanjing, China) and quantified using the Quant-iT PicoGreen dsDNA Assay Kit (Invitrogen, Carlsbad, CA, USA). After the individual quantification step, amplicons were pooled in equal amounts, and pair-end 2 × 250 bp sequencing was performed with the Illlumina NovaSeq 6000 platform at Shanghai Personal Biotechnology Co., Ltd. (Shanghai, China). The raw reads in this study have been submitted to the NCBI Sequence Read Archive (SRA) database and are available under the PRJNA818254.

### 2.4. Sequence Processing

Microbiome bioinformatics were performed with QIIME2 (ver. 2020.11) [38]. Briefly, raw sequence data were demultiplexed using the demux plugin following by primers cutting with the cutadapt plugin [39]. Sequences were then quality filtered, denoised, merged and chimera removed using the DADA2 plugin [40], resulting in Amplicon Sequence Variant (ASV) abundance tables. To remove the differences in sequencing depth, the ASV abundance tables were rarefied to the smallest sample size, which were 12,955 (bacteria via 16S rRNA) and 22,294 (protists via 18S rRNA) reads per sample. Rarefaction analyses were conducted using the diversity plugin in order to investigate the degree of sample saturation. Alpha diversity indices including Observed OTUs, Pielou’s evenness [41] and Shannon diversity [42] were calculated based on ASVs using the diversity plugin in QIIME2. Taxonomy was assigned to ASVs using the classify–sklearn naive Bayes taxonomy classifier in the feature-classifier plugin [43] against the SILVA v. 138 (for bacteria) and Protist Ribosomal (PR2) (for protists) Database, respectively. All features annotated as chloroplast and mitochondria were removed. In addition, Metazoa, which were classified as multicellular organisms, were also excluded. Non-singleton ASVs were aligned with mafft [44] and used to construct a phylogeny with fasttree2 [45].

### 2.5. Statistical Analysis

The sampling station map was plotted with Ocean Data View (ODV; https://odv.awi.de) (accessed on 2 June 2020) v. 5.3.0 [46]. All statistical analyses were conducted in R (http://www.R-project.org) (accessed on 10 October 2020) with the ‘vegan’ package [47] unless stated otherwise, and ‘ggplot2’ for graphical representations. Correlations between environmental parameters were calculated with the Pearson correlation coefficient using the ‘Hmisc’ package and were considered significant if *p* < 0.05 [48]. To explore the similarity of microbial community structures among all samples, beta diversity analysis was performed using Bray–Curtis distance [49] based on the taxonomy table. UPGMA (Unweighted Pair Group Method with Arithmetic Means) hierarchical clustering based on Bray–Curtis dissimilarity was conducted [50], and the clustering result was then used as the basis for sample grouping. Simple and partial Mantel tests (9999 permutations) were conducted to investigate the relationships between microbial community dissimilarity and environmental factors. Hierarchical Partitioning (HP) analysis with the ‘rdacca.hp’ package was performed to explore environmental components of community variation [51]. Non-metric multidimensional scaling (NMDS) analyses were also applied to reveal the significant differences between clustering groups, and the significances were evaluated by analysis of similarity (ANOSIM) [52]. To test the significance of the difference of alpha diversity between clustering groups, Student’s *t* test or Wilcoxon test with ‘ggsignif’ package were performed according to whether the data conformed to the normal distribution.

### 2.6. Function Prediction

Phylogenetic Investigation of Communities by Reconstruction of Unobserved States 2 (PICRUSt2) [53] was used to predict metagenome functions from16S rRNA gene data, and the metagenomes were aligned to the Kyoto Encyclopedia of Genes and Genomes (KEGG) bioinformatics database. The Nearest Sequenced Taxon Index (NSTI) score was determined for each sample to evaluate the prediction accuracy of PICRUSt [54], and a lower value (roughly < 0.06) indicated that the functional profiles obtained were closely related to the reference genome. PCA was employed to group or separate samples using ImageGP [55] based on the function abundance of KEGG pathways on three levels. Significantly different KEGG pathways on level II between two salinity clusters were determined by Statistical Analysis of Metagenomic Profiles (STAMP) [56], using two-sided Welch’s *t* test with a Storey (normal distribution) or Benjamini–Hochberg (abnormal distribution) FDR multiple testing correction [57] (adjusted *q* value < 0.05).

## 3. Results

### 3.1. Environmental Characteristics

The salinity in the entire study area ranged from 0 to 32.1 with an increasing gradient from Changjiang River to the ocean (Figure 1, Table S1), reflecting significant effects of freshwater runoff. The stations in this study were distributed onto two sections. Section C is about 431.93 km long from the Changjiang River to the outer sea. It is mainly affected by the Changjiang Diluted Water, characterized with large gradients of salinity (0~32.1) and nutrients (DIN: 1.00~117.72 μM; DIP: 0.03~1.62 μM; DSi: 2.36~118.17 μM). Section Y extends eastward from the near shore of Zhoushan, spanning a geographical distance of 148.33 km. It is less influenced by runoff water and a variation of salinity (27.8–30.6) and nutrients (DIN: 1.74~26.90 μM; DIP: 0.05~0.83 μM; DSi: 0.51~24.38 μM) (Figure 1 and Table S1), as this section is smaller compared with section C. Generally, nutrient contents decreased gradually from the river to ocean or from coast to ocean. Moreover, there was extremely significant negative correlation between salinity and DIN (*r* = −0.98, *p* < 0.001), DSi (*r* = −0.97, *p* < 0.001), as well as DIP (*r* = −0.55, *p* < 0.01) (Table S2). Surface water temperature ranged from 26.39 to 29.46 °C and had no significant correlation with any other environmental parameters. SPM had a maximum content at station C7, which was 1.14 g L^−1^, and it was much higher than the values of other samples.

### 3.2. Spatial Patterns in Bacterial and Protistan Community

A total of 16,807 bacterial ASVs and 6132 protistan ASVs were obtained from 27 surface water samples. After sequence rarefying, 12,955 and 22,294 reads were yielded for each bacterial and protistan sample, respectively. Although some rarefaction curves did not reach an asymptote, the sequencing effort detected the majority of both bacterial and protistan species, which indicated that the sequencing depth was sufficient to represent the total diversity (Figure S1).

**Figure 2:**
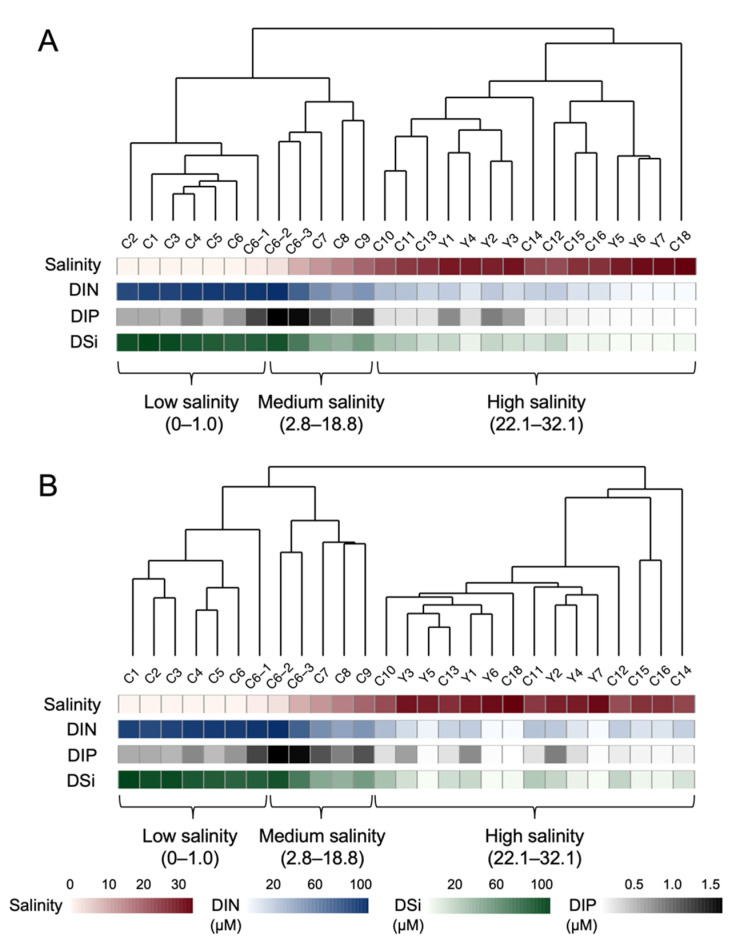
UPGMA clustering based on bacterial (**A**) and protistan (**B**) plankton community composition similarities. Samples were collected from surface water along two west–east transects of the Changjiang Estuary and its adjacent area with pronounced salinity gradient. Both bacterial and protistan plankton communities were clustered into three salinities: low-salinity cluster (0–1.0), medium-salinity cluster (2.8–18.8) and high-salinity cluster (22.1–32.1). Color stripes represent the values of four environmental parameters at each station.

The bacterial and protistan ASVs were assigned to 2081 and 772 species, respectively (Datasheet S1 and S2). UPGMA analysis showed that both bacterial and protistan communities could be divided into three clusters according to the same salinity ranges (Figure 2). The first cluster included the western stations C1–C6-1 (7 stations), with the salinity range from 0 to 1. The second cluster included stations C6-2–C9 (5 stations), with the salinity range from 2.8 to 18.8. The third cluster included the rest 15 stations, covering the salinity range from 22.1 to 32.1.

The results of the simple Mantel test demonstrated that both bacterial and protistan community dissimilarity were significantly correlated with salinity, DIN, DIP, DSi, SPM, pH, integrated environmental factors and geographical distance (Table 1 and Table S3). The correlation between the community composition and the set of all environmental parameters (Euclidean distance of all environmental parameters) (*r* = 0.780 for bacterial community and 0.703 for protistan community) was greater than that of the geographical distance (*r* = 0.707 and 0.513, respectively). In particular, after conducting the partial Mantel test, the correlation coefficient between environmental variables and community dissimilarity was much higher than that between geographical distance and community dissimilarity, indicating that environmental factors had greater effects on community dissimilarity than geographical distance did. Salinity, DIN and DSi had the highest *r* values, suggesting that they might be the key factors in shaping the community structures of both bacterial and protistan communities. Compared with protists, all factors exhibited a slightly greater relevance with the bacterial community variation, except for SPM.

Because DIN and DSi decreased linearly with salinity along the sampling transect C (Table S2), the partial Mantel test was performed to distinguish the effects of determinate factors. It can be found that the correlation coefficient decreased a lot when the effect of certain variables was controlled. The correlations between bacterial community dissimilarity and salinity differences were still significantly high after controlling for DIN and DSi, which were 0.421 (*p* < 0.001) and 0.497 (*p* < 0.001), respectively (Table 1). While the correlations between bacterial community dissimilarity and DIN/DSi differences were much lower when controlling for salinity, *r* = 0.249 (*p* < 0.001) and *r* = 0.267 (*p* < 0.01). The partial Mantel test also revealed that salinity still strongly correlated with changes in protistan taxonomic composition when controlled for DIN (*r* = 0.496, *p* < 0.001) and DSi (*r* = 0.517, *p* < 0.001). However, the correlation between protistan community dissimilarity and DIN/DSi significantly decreased after controlling for salinity, which was 0.056 (*p* > 0.05) and 0.139 (*p* < 0.05), respectively (Table 1).

**Figure 3:**
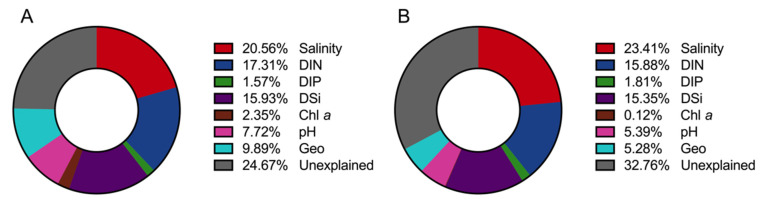
Pie charts for the independent explanation of environmental factors by hierarchical partitioning. The percentage value was the proportion of the variation explained by each environmental factor to the total variation of bacterial (**A**) and protistan (**B**) communities.

In order to quantitatively evaluate the independent explanation of environmental factors on community structures, hierarchical partitioning analysis was performed (Figure 3). The total explanation rates (*R^2^*) of environmental parameters to changes in taxonomic composition of bacterial and protistan communities were 75.33% and 67.24%, respectively. Among them, salinity had the largest contribution to the composition variation of bacterial and protistan communities, accounting for 20.56% and 23.41%, respectively, followed by DIN (17.31% and 15.88%) and DSi (15.93% and 15.35%). The unexplainable part showed that there were other factors affecting the community composition but not in a consistent and predictable manner.

**Figure 4:**
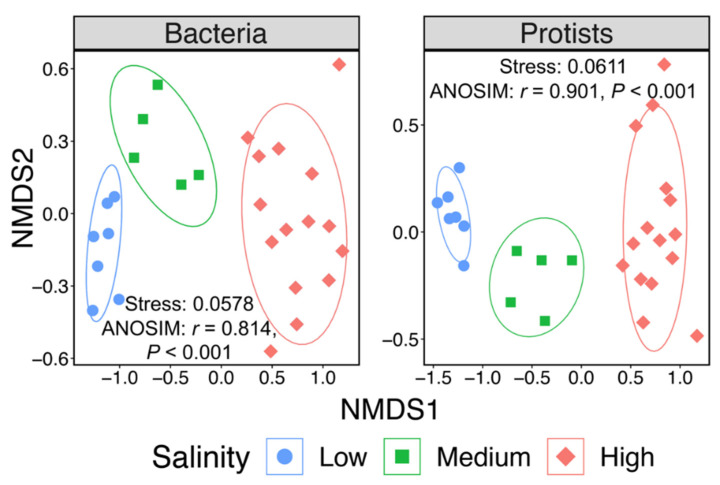
Nonmetric multidimensional scaling (NMDS) plots based on bacterial and protistan beta-diversity dissimilarity (Bray–Curtis). The circle (blue), square (green) and diamond (red) represent sampled sites of low-salinity cluster (*n* = 7), medium-salinity cluster (*n* = 5) and high-salinity cluster (*n* = 15), respectively.

UPGMA clustering and determinate factor analyzing suggested that salinity was the major structuring factor for both bacterial and protistan community structures in surface water in the Changjiang Estuary rather than geographic distance or any other environmental factor. Therefore, 27 Changjiang Estuary surface water stations were clustered into three salinity groups based on UPGMA clusters, which were low-salinity cluster (low: salinity 0–1.0), medium-salinity cluster (medium: salinity 2.8–18.8) and high-salinity cluster (high: salinity 22.1–32.1). NMDS analyses of both bacterial and protistan communities showed the same clustering patterns as the UPGMA analyses (Figure 1 and Figure 4). The stress values were both less than 0.1, indicating that the result of NMDS analyses had good explanatory significance. ANOSIM also showed that there were significant differences between different salinity clusters (*r* = 0.814, *p* < 0.001 and *r* = 0.901, *p* < 0.001, for bacterial and protistan community, respectively) and the dissimilarity of the bacterial and protistan communities was higher between different salinity clusters than it was between samples within the same salinity cluster (Figure S2).

### 3.3. Alpha-Diversity Patterns

**Figure 5:**
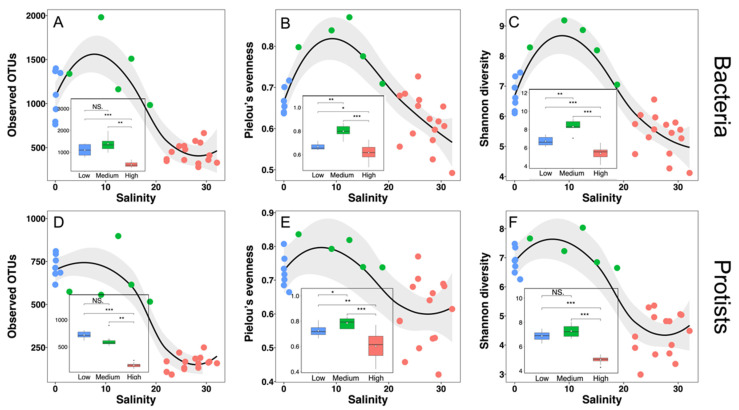
Variation of three alpha diversity indices of bacteria (**A**–**C**) and protists (**D**–**F**). Scatter plots displayed the three diversity indices as a function of salinity, and the shaded area is the 95% confidence interval of the smooth-fitting curve. Box plots showed the distribution of each index in low-salinity cluster (*n* = 7), medium-salinity cluster (*n* = 5) and high-salinity cluster (*n* = 15), and the white diamond represents the mean value of each set of data. According to whether the data conformed to the normal distribution, the differences of each alpha diversity index between the two clusters were tested using Student’s t test or Wilcoxon test. * *p* < 0.05; ** *p* < 0.01; *** *p* < 0.001; NS, not significant.

To evaluate the effect of salinity on alpha-diversity of Changjiang Estuary bacterial and protistan communities, Observed OTUs, Pielou’s evenness and Shannon index were calculated. Compared with alpha-diversity in low-salinity and medium-salinity waters, all three alpha-diversity indices in both bacterial and protistan communities were significantly decreased in high-salinity waters (*p* < 0.05) (Figure 5). The Observed OTUs of the high-salinity bacterial community were 436.33 ± 112.20 (*n* = 15), which was only 39.60% of the low-salinity ones (1101.86 ± 275.78, *n* = 7) (Figure 5A). The decrease in alpha-diversity from low-salinity waters to high-salinity waters was more pronounced in the protistan community compared with the bacterial community. The Observed OTUs of the high-salinity protistan community (721.71 ± 69.32) were only 22.29% of the low-salinity ones. Although Pielou’s evenness of bacterial and protistan communities in high-salinity waters decreased only 7.58% and 16.90%, respectively, comparing with those in low-salinity waters, it was statistically different (*p* < 0.05) (Figure 5B,E). The Shannon diversities of high-salinity bacterial and protistan communities were 80.21% and 63.66% of those in low-salinity waters, respectively (Figure 5C,F). Differences in the effect of salinity on alpha-diversity between bacterial and protistan communities were also observed. The highest bacterial diversity (Shannon diversity) was identified in medium-salinity waters (Figure 5C), while there was no significant difference in Shannon diversity between low-salinity and medium-salinity protistan communities (*t* test, *p* =0.219) (Figure 5F).

### 3.4. Community Composition

**Figure 6:**
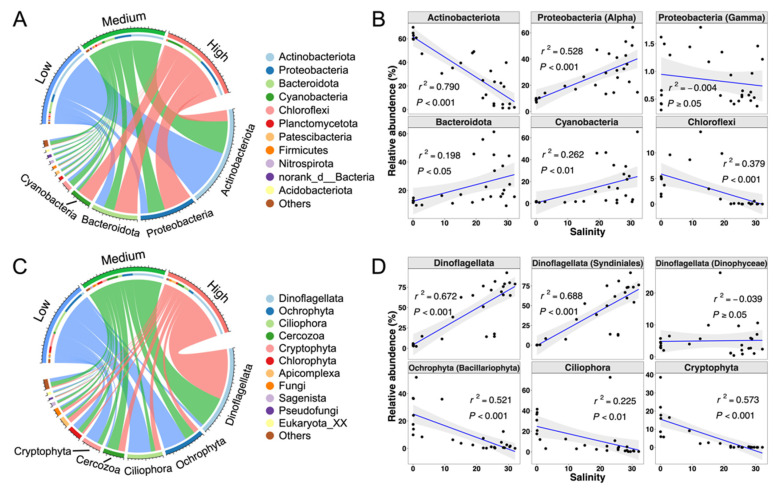
The chord diagrams indicate the mean relative abundance of bacteria (**A**) and protists (**C**) at the phylum level of the low-salinity cluster, medium-salinity cluster and high-salinity cluster. Scatter plots display the relative abundance of some representative phyla (class) of bacteria (**B**) and protists (**D**) as a function of salinity. The blue line is the linear regression fit and the shaded area is the 95% confidence interval. *r^2^* and *p* represent the goodness of fit and significance, respectively.

**Figure 7:**
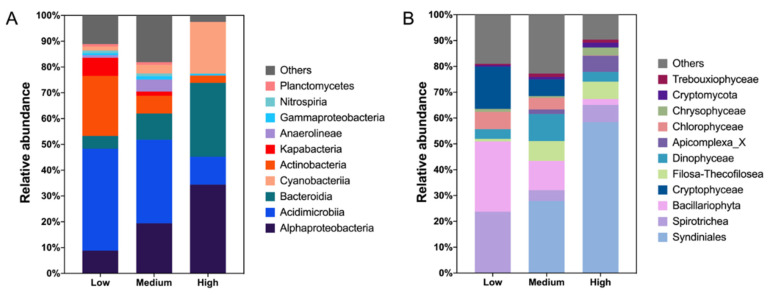
Average relative abundance of bacteria (**A**) and protists (**B**) at the class level in low-salinity cluster (*n* = 7), medium-salinity cluster (*n* = 5) and high-salinity cluster (*n* = 15). Relative abundance of less than 1% was assigned to Others.

A total of 54 bacterial phyla were identified from all samples, where Actinobacteriota, Proteobacteria, Bacteroidota and Cyanobacteria were the dominant taxa at the phylum level (Figure 6A, Datasheet S1). Among them, Actinobacteriota accounted for 31.39% of the total sequences, and it was mainly distributed in low-salinity waters. Its abundance decreased with increasing salinity (Figure 6A,B). Proteobacteria accounted for 25.81%, and its abundance was the highest in the high-salinity cluster. In the phylum Proteobacteria, Alphaproteobacteria was the top contributor (24.99%), and the relative abundance increased with increasing salinity (*p* < 0.001), while the relative abundance of Gammaproteobacteria was extremely low in the entire study area (Figure 6B, Figure 7A and Figure S3A). Bacteroidota accounted for 21.60% of all sequences, while the correlation between relative abundance of Bacteroidota and salinity was significantly positive (*p* < 0.05, Figure 6B). The higher relative abundance of Cyanobacteria was observed in some high-salinity samples, while it was low in lower-salinity samples (Figure 6A,B).

A total of 39 protistan phyla were identified from all stations, with Dinoflagellata, Ochrophyta, Ciliophora and Cercozoa being the dominant phyla (Figure 6C, Datasheet S2). Dinoflagellata accounted for 42.72% of all sequences, followed by Ochrophyta, Ciliophora and Cercozoa, accounting for 13.46%, 12.45% and 7.92%, respectively. The other groups, including Cryptophyta, Chlorophyta, Apicomplexa and Fungi, contributed 17.79% of all sequences. The main categories of protists had various distribution characteristics along the salinity gradient. Dinoflagellata was mainly found in brackish and marine water, but with extremely low abundance in fresh waters (Figure 6C), while the relative abundance of Dinoflagellata was positively correlated with salinity (*p* < 0.001) (Figure 6D). However, Ochrophyta, Ciliophora, and Cryptophyta had the opposite distribution patterns, with higher relative abundance in low-salinity waters. In particular, Cryptophyta was almost not observed in high-salinity waters. The relative abundance of Ochrophyta, Ciliophora, and Cryptophyta were negatively correlated with salinity (*p* < 0.01) (Figure 6D and Figure S4). Cercozoa, Apicomplexa and Fungi showed no significant change in relative abundance with the salinity gradient (*p* > 0.05) (Figure S4).

The dominant phylum of protists was Dinoflagellata, which was mainly contributed by class Syndiniales and class Dinophyceae. Syndiniales and Dinophyceae accounted for 88.17% and 11.73% of the entire sequences of the phylum Dinoflagellata, respectively (Figure 7B and Figure S3B, Datasheet S2). These two classes accounted for 37.67% and 5.01% of the total protistan sequences, respectively. The abundance of Syndiniales was extremely low in low-salinity waters, while it increased with increasing salinity (*p* < 0.001) (Figure 6D and Figure 7B). However, there was no significant correlation between Dinophyceae and salinity (*p* > 0.05). Class Bacillariophyta contributed 77.30% of all sequences belonging to Ochrophyta, which took up 10.40% of all protistan sequences (Datasheet S2, Figure S3B). Bacillariophyta exhibited a characteristic of decreasing abundance with increasing salinity, and its abundance in high-salinity water was extremely low (*p* < 0.001) (Figure 6D and Figure 7B).

### 3.5. Profiles of Bacterial Functional Prediction

**Figure 8:**
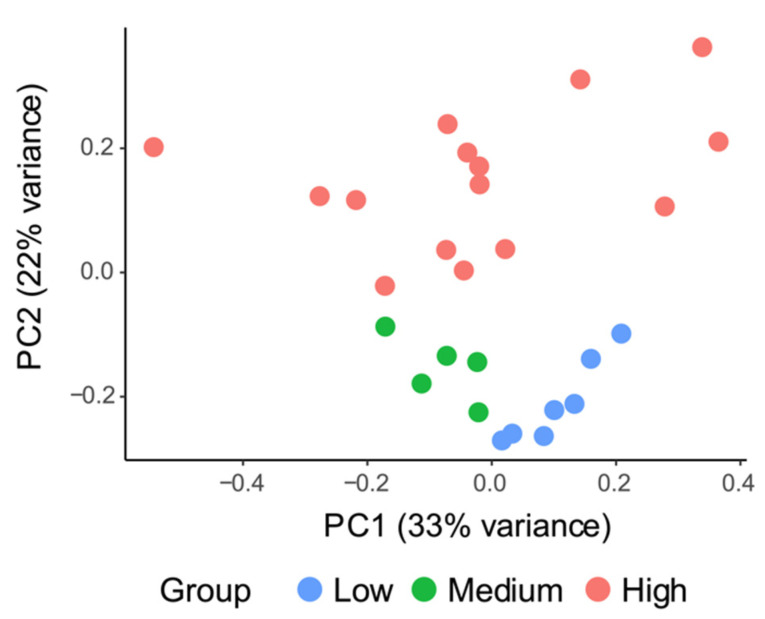
PCA plot based on gene functional abundance on KEGG level II predicted by PICRUSt2. Each point represents a sample, and the color blue, green and red represent the sample groups of low-, medium- and high-salinity, respectively.

PICRUSt2 was used to predict metagenome functions from 16S rRNA gene data and was categorized by KEGG classification on three different levels of KEGG pathways. In our study, the NSTI scores ranged from 0.027 to 0.130 with an average of 0.067 ± 0.027 (mean ± SD, *n* = 27) (Table S4). The PCA plot based on gene functional abundance (Datasheet S3) on level I (Figure S5A) indicated that the functional distribution characteristics of the samples in low-salinity waters were more similar, while those of the samples in high-salinity waters showed higher diversity. However, the biological functions of the three clusters were significantly segmented on level II (Figure 8) and III (Figure S5B), indicating significant differences in gene function.

Function prediction revealed 37 pathways on KEGG level II. Amino acid metabolism, carbohydrate metabolism, and metabolism of cofactors and vitamins were primary predictive pathways at KEGG Level II (Table S5). Among the 37 pathways, 24 had significant differences between low- and medium-, 26 had significant differences between low- and high-, and 12 had significant differences between medium- and high-salinity clusters. Amino acid metabolism (14.11%), metabolism of cofactors and vitamins (12.38%), and glycan biosynthesis and metabolism (2.80%) were the highest categories in the high-salinity cluster and did not differ between the low- and medium-salinity clusters. Metabolism of other amino acids (8.11%) and cell growth and death (1.47%) were the highest in the high-salinity cluster, followed by the medium-salinity cluster, and were lowest in the low-salinity cluster.

## 4. Discussion

### 4.1. Contribution of Salinity and Other Environmental Factors in Shaping Microbial Communities

In this study, a total of 27 sampling sites (Figure 1) of two distinct sections (section C and section Y) were selected for research. Our sampling stations covered a salinity range of 0 to 32.1, with a DIN content range of 1.00 to 117.72 μM. Although the geographic location and the environmental gradient were different between two sections (Table S1), microbial community structure was mainly determined by salinity, based on the UPGMA cluster (Figure 2), simple and partial Mantel test (Table 1 and Table S3), HP analysis (Figure 3) and NMDS analysis (Figure 4). This result correlated strongly with previous studies, which showed that salinity is a major global driver of prokaryotic community structure [4,58]. Moreover, salinity had a greater effect on the protistan community structure than on the bacterial community structure based on partial Mantel test and HP analysis (Table 1, Figure 3). The simple and partial Mantel test (Table 1 and Table S3) and HP analysis (Figure 3) suggested that besides salinity, nutrients and geographic distance played roles in shaping microbial community structures, which was consistent with other research studies in the river–ocean continuum [11,59] and coastal transects [9].

Our study implied that bacterial and protistan community structures of 27 sampling stations were both clustered into three groups according to the same salinity range, namely 0–1.0, 2.8–18.8 and 22.1–32.1. This conclusion was not consistent with previous studies in the Baltic Sea, which indicated that the salinity borders for bacterial and protistan community structure were different [19,20]. The border between the brackish-marine and the brackish bacterial cluster coincides with the Darss Sill (salinity ~9) [60], while salinity of 10–12 was identified as the strongest environmental salinity barrier for the protistan community in the Baltic Sea [19,20]. Although the sampling area and salinity range in those studies had some overlap, the sampling time and sampling sites were not exactly the same. Therefore, it is not a perfect example to compare the response of bacterial and protistan community structures to salinity change, and more research needs to be conducted. The interaction between bacteria and protists in the marine environment has been widely revealed, including predation, symbiosis and parasitism [61,62,63]. The ubiquitous co-occurrence relationship between bacteria and protists may account for our results that both bacteria and protists communities were clustered into the same salinity group. However, co-occurrence network analysis should be carried out for further explanation.

Differences were observed between the effects of other environmental factors on bacterial community structure and those on protistan community structure. After controlling for the influence of salinity, DIN and DSi still had significant correlation with bacterial community composition, while no significant correlations between DIN/DIP and the composition of the protistan community existed (Table 1). Furthermore, the correlation between DSi and protistan community dissimilarity was still significant after controlling for salinity. This difference might be explained by the characteristics of community composition. A high proportion of protists, including Syndiniales, Spirotrichea and Filosa-Thecofilosea (Figure 7B and Figure S3B, Datasheet S2), which contributed 53.61% of total protistan sequences, was parasitic or predatory [64,65] and did not directly rely on the availability of DIN and DIP in the surface water. In contrast, another important protist, Bacillariophyta, which contributed 10.40% of total protistan sequences, required DSi for its growth. This may account for why DSi had a significant impact on the protistan community structure as mentioned above.

### 4.2. Microbial Alpha Diversity and Microbial Community Composition Structured by Salinity

Not only were the microbial community structures determined by salinity, salinity also affected microbial alpha diversity. Similar to what was observed in other estuaries [10,20,66,67], the alpha diversity indices of both bacterial and protistan communities were significantly decreased in high-salinity waters in this study. The microorganisms living in saline waters adopted two distinct strategies to cope with osmolarity changes, one of which was the synthesis or uptake of organic compatible solutes that function as osmoprotectants [68]. The enriched predicted functions in high-salinity bacterial communities were involved in the mechanisms of different organic compatible solutes, e.g., amino acid metabolism (14.12%), metabolism of cofactors and vitamins (12.38%), metabolism of other amino acids (8.11%), glycan biosynthesis and metabolism (2.80%) (Table S5). Synthesis or uptake of organic compatible solutes requires extra energy consumption; therefore, the growth of microbes in high-salinity waters in this study, which was also nutrient-limited environment, must be inhibited. Moreover, similar to the Mississippi river and the Columbia river, the Changjiang River has large runoff with rich nutrients that can support more microbial species in fresh and brackish waters [69].

However, different patterns have been observed in previous planktonic studies [11,19,21,70]. The studies of the Baltic Sea showed no clear trend in alpha diversity for bacterial or microeukaryotic communities along the salinity gradient (from 0 to 35) [19,21,70]. The studied area in the Baltic Sea has different hydrography from our sampling area, due to its negligible tidal activities and relatively high stable hydrography [71]. Unlike the Baltic Sea, our study area does not have sufficient residence time for the establishment of autochthonous salt-adapted microbial plankton communities [19]; therefore, it could not further support increases of alpha diversity in the high-salinity waters. Furthermore, the study along the Waiwera river–estuary–sea transect showed that the Shannon index of planktonic bacteria and microeukaryotes increased in brackish and marine water versus in freshwater [11]. This transect had an increase in dissolved reactive phosphorus with increasing salinity; thus, high nutrient supply might provide energy for the synthesis or uptake of organic compatible solutes, and it promoted high alpha diversity in brackish and marine water along the Waiwera river–estuary–sea transect, which was opposite from our observation of nutrients decreasing with increasing salinity in the Changjiang Estuary.

Remane’s ‘Artenminimum’ concept [18], which proposes minimum diversity for benthic macrofauna in the brackish horohalinicum (salinity 5–8), was challenged for bacteria [19,72] and microeukaryotes [11] in the previous studies, including in this study. We observed no decline in both bacterial and protistan alpha diversity in all brackish stations (salinity 2.8–18.8); thus, the planktonic microbial diversity does not follow Reman’s concept. This is consistent with previous reports of Columbia River, suggesting that a river with large runoff may act as a seed bank for microbial diversity when mixed with seawater in the estuaries [10,73]. This might be partially explained by the highly debated intermediate-disturbance hypothesis [74], which suggests that an ecosystem under mild disturbance would have maximal community diversity.

Distinct patterns between bacterial and protistan communities have been observed, that is, the Shannon diversity of bacterial community was the highest in the medium-salinity waters, while there was no significant difference in Shannon diversity of the protistan community between low- and medium-salinity waters (Figure 5C,F). This medium-salinity aera was where freshwater and seawater exchanged rapidly; thus, this microbial community consisted of species from both end members. The relative abundance of each taxonomic group at the bacterial phylum level changed gradually along three salinity groups, exhibiting a mixture of freshwater and seawater species. In addition, high SPM content brought by Changjiang River runoff can act as a source of organic matter and serve as a nutrient source for bacteria, supporting high diversity in this area. However, the saline boundaries for eukaryotic microbes were ubiquitous [75,76], as they were distributed only in a certain salinity range. Furthermore, many protists are parasitic in multicellular organisms, which were reported as having the lowest diversity in brackish water (Remane’s ‘Artenminimum’ concept); thus, the diversity of parasitic protistan was affected by the distribution of their hosts. At last, high turbidity in the medium-salinity area inhibited the growth of phytoplankton from both freshwater and seawater. In conclusion, alpha diversity of bacteria and protistan communities in medium-salinity waters was affected by different mechanisms. However, unexpected extremely high observed OTUs of the protist community were observed in station C7 (Figure 5D). This station is located in the Changjiang Estuarine turbidity maximum zone (TMZ), with the highest SPM content among all sampling stations. Due to the strong vertical mixing in TMZ, the bottom water and sediment were resuspended in the sea surface, with planktonic and benthic protists mixed together in C7, resulting in the highly observed OTUs.

In accordance with previous studies along estuarine salinity gradients, typical phylum/class occupied different salinity ranges [19,77], e.g., relative abundance of Actinobacteria decreased with increasing salinity, while that of Alphaproteobacteria showed the opposite trend. However, regional differences lead to inconsistent patterns of Bacteroidota and Cyanobacteria in different research studies [10,19,77]. Moreover, Gammaproteobacteria was not the major class in this survey area, which was inconsistent with many previous studies [19,32,77]. The seasonal pattern of Gammaproteobacteria in the Bohai Sea, reported by Wang et al. [78], which was consistent with our winter data (data not shown), might be the reason. Lack of Chloroflexi in high-salinity waters in our study was in accordance with the hypothesis that origins of pelagic Chloroflexi are likely from soil and sediment habitats, and freshwater habitats harbor the most diverse phylogenetic assemblages in comparison to brackish and marine habitats [79]. In our study, the most significant effect of salinity on the distribution of protists was that Syndiniales only appeared at medium- and high- salinity stations. Syndiniales are distributed widely with high abundance in the oceans worldwide [7,64] and were also the second-dominant protistan order in the study of coast waters of China, including the Changjiang Estuary and the East China Sea [7]. Salinity 1.0 represented a salinity barrier that was difficult to cross for Syndiniales, as it was only distributed in waters with a salinity greater than 1.0. Contrary to Syndiniales, zooplankton Ciliophora dominated in low-salinity waters, mainly due to the major class Spirotrichea in the survey area, which preferred freshwater and brackish conditions, similar to what was observed in some previous studies [80,81].

As expected, the most important primary producer in this region was Bacillariophyta, dominated by *Skeletonema*, especially in the low-salinity waters [82,83], showing negative correlation between relative abundance and salinity. *Skeletonema* was generally found in the Changjiang diluted water plume where the salinity is lower than 23 [82], which demonstrates the important roles of salinity in regulating community structure. Cryptophyta (mainly class Cryptophyceae) also accounted for a large proportion of photosynthetic phytoplankton in low- and medium-salinity waters in the Changjiang Estuary, which was consistent with previous studies showing that the typical distribution of Cryptophyta was from freshwater to brackish conditions [21,84]. The distribution of most bacteria and protists occupied a specific salinity range, which reflected their adaptability to the environment.

Using the 18S rRNA gene as a barcode to investigate protistan diversity is widely accepted [85,86]. However, the biggest disadvantage of using this method is that the 18S rRNA gene copy numbers vary in different phylogenetic groups. More copy numbers of the 18S rRNA gene have been found in dinoflagellates and ciliates, due to large cell volumes and vast genomes [87,88]. Thus, dinoflagellates and ciliates might be overestimated, compared with the smaller flagellates, such as prasinophytes. High relative abundance of dinoflagellates in this study might be related to its high copy numbers of the 18S rRNA gene. Still, the variation in relative abundance of the same taxonomic group across samples is still reliable, allowing for us to test for correlations with environmental variations.

## 5. Conclusions

Across the Changjiang Estuary to ocean transect, salinity was the primary determinant for microbial community structures. DIN and DSi had distinct effects on bacterial and protistan community dissimilarity. Alpha diversity of bacterial and protistan communities both decreased in the higher salinity waters, while they had distinct trends in medium-salinity waters. Microbial community composition was also affected from the estuary to the adjacent areas mainly by salinity. Overall, our study demonstrated salinity as the primary impact on both bacterial and protistan community structure, alpha diversity, and beta diversity, while different patterns affected by salinity were revealed between bacterial and protistan communities.

## Figures and Tables

**Table 1:** Simple and partial Mantel tests for the correlations between bacterial/protistan community dissimilarity and salinity, DIN, DSi and geographical distances based on Spearman rank correlation coefficient. DIN, dissolved inorganic nitrogen; DSi, dissolved silica; Geo, geographical distance. The significance was tested based on 9999 permutations, and *p* value < 0.05 was considered as a significant effect (* *p* < 0.05; ** *p* < 0.01; *** *p* < 0.001).

	Simple Mantel Test			Partial Mantel Test	
	Bacteria	Protists		Bacteria	Protists
Effects of	*r*	*r*	Control for	*r*	*r*
Salinity	0.884 ***	0.864 ***	DIN	0.421 ***	0.496 ***
			DIP	0.875 ***	0.857 ***
			DSi	0.497 ***	0.517 ***
			Geo	0.778 ***	0.819 ***
DIN	0.866 ***	0.816 ***	Salinity	0.249 ***	0.056
			DIP	0.859 ***	0.811 ***
			DSi	0.308 ***	0.205 **
			Geo	0.754 ***	0.740 ***
DIP	0.263 **	0.226 **	Salinity	0.048	−0.02
			DIN	−0.15	−0.162
			DSi	−0.077	−0.11
			Geo	0.208 **	0.167 *
DSi	0.854 ***	0.814 ***	Salinity	0.267 ***	0.139 *
			DIN	0.144 *	0.174 *
			DIP	0.843 ***	0.805 ***
			Geo	0.719 ***	0.740 ***
Geo	0.707 ***	0.513 ***	Salinity	0.313 ***	−0.198
			DIN	0.370 ***	−0.049
			DIP	0.697 ***	0.495 ***
			DSi	0.328 ***	−0.098

## Data Availability

The raw reads in this study are available under the PRJNA818254 from NCBI Sequence Read Archive (SRA) database.

## Supplementary Materials

The following supporting information can be downloaded at: https://www.mdpi.com/article/10.3390/microorganisms10050991/s1, Figure S1: Rarefaction curve of bacterial (A) and protistan (B) sequencing; Figure S2: Analysis of similarities (ANOSIM) between different salinity groups of bacteria (A) and protists (B); Figure S3: Community composition of bacteria (A) and protists (B) at class level in each sample. Species with low abundance were assigned to ‘Others’ (at least less than 1%); Figure S4: Scatter plots showing the relative abundance of some representative phyla (class) of protists as a function of salinity. Blue line, linear regression fit; shaded area, 95% confidence interval. *r^2^* and *p* represent goodness of fit and significance, respectively; Figure S5: PCA plots based on gene functional abundance on level I (A) and III (B) of KEGG predicted by PICRUSt2. Each point represents a sample, and the color blue, green and red represent the salinity groups of low, medium and high, respectively; Table S1: Environmental parameters. DIN, dissolved inorganic nitrogen; DIP, dissolved inorganic phosphorus; DSi, dissolved silica; Chl *a*,c hlorophyll *a*; SPM, suspended particulate matter; Table S2: Pearson correlation coefficient between environmental variables. ‘*’ represents the significance level (* *p* < 0.05; ** *p* < 0.01; *** *p* < 0.001). Bold face values indicate significant correlations at *p* < 0.05; Table S3: Results of simple and partial Mantel tests demonstrating spearman’s correlations of environmental factors (Enclidean), total environmental variability (Euclidean) and geographic distance with bacterial and protistan beta-diversity dissimilarity (Bray–Curtis) against all sampled sites (*n* = 27, with 9999 permutations). Env refers to the Euclidean distance of the set of all environmental parameters including salinity, temperature, DIN, DIP, DSi, Chl *a*, SPM and pH. Geo refers to the geographic distance. *r*_env_ refers to the correlation coefficient between abiotic drivers and microbial dissimilarity controlled by the set of all environmental parameters derived from Mantel test. *r*_geo_ refers to the correlation coefficient between abiotic drivers and microbial dissimilarity controlled by geographic distance derived from partial Mantel tests. * *p* < 0.05; ** *p* < 0.01; *** *p* < 0.001; Table S4: The Nearest Sequenced Taxon Index (NSTI) scores calculated from PICRUSt2 of each sample; Table S5: The relative abundance of KEGG pathways at the second level and differences between each two groups with different salinity (*t* test, * *p* < 0.05; ** *p* < 0.01; *** *p* < 0.001); Datasheet S1: Feature table of bacteria; Datasheet S2: Feature table of protists; Datasheet S3: Gene functional abundance predicted by PICRUSt2 on three levels.
